# Percutaneous Thrombectomy in Patients with Occlusions of the Aortoiliac Segment: A Case Series

**DOI:** 10.1007/s00270-022-03222-y

**Published:** 2022-08-24

**Authors:** Malte Maria Sieren, Julian Pfarr, Schekeb Aludin, Karim Mostafa, Erik Stahlberg, Franz Wegner, Sam Mogadas, Rene Rusch, Marco Horn, Philipp Schäfer

**Affiliations:** 1 Department of Interventional Radiology, University Hospital Schleswig-Holstein, Campus Lübeck, Ratzeburger Allee 160, 23562 Lübeck, Germany; 2 Department of Radiology and Nuclear Medicine, University Hospital Schleswig-Holstein, Campus Luebeck, Lübeck, Germany; 3 Department of Radiology & Neuroradiology, University Hospital Schleswig-Holstein, Campus Kiel, Kiel, Germany; 4 Department of Vascular Surgery, University Hospital Schleswig-Holstein, Campus Luebeck, Lübeck, Germany; 5 Department of Vascular Surgery, University Hospital Schleswig-Holstein, Campus Kiel, Kiel, Germany

**Keywords:** Percutaneous thrombectomy, Aortic occlusion, Iliac occlusion, Endovascular procedures

## Abstract

**Objective:**

Thrombectomy of the aortoiliac segment remains a challenge for surgical and endovascular revision. This study aimed to evaluate the concept of percutaneous thrombectomy in patients with aortoiliac segment occlusions.

**Materials & Methods:**

Eighteen patients with aortoiliac occlusion who underwent percutaneous thrombectomy were retrospectively identified using the local picture archive and divided into the stent-graft (*N* = 10) and native vessels (*N * =  8) groups. The procedure was performed by placing a 12–24 French sheath adjacent to the distal end of the occluded vessel segment. The occlusion was passed with a balloon catheter which was retracted after inflation, to deliver the thrombus into the sheath. Technical success (reperfusion of the vessel and no residual thrombus/stenosis < 30%), complications and primary arterial patency were assessed. Follow-up included computed tomography angiography and evaluation of the clinical situation via telephone.

**Results:**

Technical success was achieved in 38% (7/18) of patients after percutaneous thrombectomy alone and in 100% after additional procedures. The most common complication was peripheral embolism (44%, 8/18), which was treated successfully in all cases and was linked to a mismatch between the sheath and target vessel of ≥ 1 mm (*P* < .01). There were no significant differences in the incidence of complications between the two groups. Primary patency was 72% (13/18) with no significant difference between groups (*P * =  .94). Follow-up CT scans were available for 13/18 patients (72%), with a mean follow-up time of 270  ±  146 days. All patients were contacted via phone (follow-up time, 653  ±  264 days).

**Conclusion:**

Percutaneous thrombectomy appears to be effective for revascularization of the aortoiliac segment, both in stent-grafts and in native vessels. The most common complication is peripheral embolism; however, the risk may be reduced by choosing an adequate sheath size.

## Introduction

Thrombectomy of vascular occlusions, especially in the aortoiliac segment, remains a challenge for surgical and endovascular revision. However, the evolution of vascular medicine has led to a plethora of available options to re-establish arterial patency, all of which inhibit certain disadvantages.

The Fogarty manoeuvre (FM) is most often used to perform thrombectomy [1]. Although this technique can be flexibly employed, it is an open surgical approach. Potential complications include bleeding and wound healing disorders, and clinical outcomes have been unsatisfactory in specific pathologies [2, 3]. With the evolution of endovascular medicine, various minimally invasive approaches such as aspiration, mechanical rotation [4, 5], and rheolytic thrombectomy [6, 7] have become available [8]. Their viability has been well-researched and they can be considered the gold standard for all vessel segments smaller than the external iliac arteries. However, the literature reports variable success rates in large thrombus volumes, as usually found in occlusions of the aorto-iliac segment [9].

A solution to this clinical dilemma might be percutaneous thrombectomy. Removal of large thrombus volumes require greater vessel access. Establishing such percutaneously has become possible owing to the routine use of vascular closure devices, which is obligatory for this procedure. However, only a few case reports have described the procedure in a clinical setting [10, 11]. Furthermore, data regarding thrombectomy procedures in the aortoiliac segment, in general, is scarce [12, 13].

Therefore, this study aimed to evaluate the technical success rate and outcome of percutaneous thrombectomy in a case series of patients who were treated for thrombotic occlusions of the aortoiliac segment.

## Material and Methods

### Study Population

Since this was a retrospective study, the local ethics committee waived the need for de novo consent. All patients consented to the medically indicated procedures as per the clinical standard.

Eighteen patients with aortoiliac occlusion who underwent percutaneous thrombectomy of the aortoiliac segment between 1 January 2017 and 31 July 2021 were retrospectively identified using a database query. The exclusion criteria for the endovascular approach were bilateral vascular occlusion and common femoral artery occlusion. The patients were divided into two groups. The first group consisted of patients with thrombosis in stent grafts [stent-graft group], and the second group comprised patients with thrombosis in native vessels [native vessels group].

In addition to demographic characteristics, peri-interventional imaging, location, and length of vessel occlusion were determined. The age of the lesion was classified as acute (< 2 weeks), subacute (> 2 weeks to < 4 weeks), or chronic (> 4 weeks) according to the onset of symptoms, as reported by the patient. For each patient, comorbidities and the degree of vessel calcification were noted [14]. Both the length of the vessel occlusion and the degree of vessel calcification were included due to their possible association with an increased risk of failure of primary thrombectomy and complications.

### Procedure Protocol

All interventions were performed by radiologists with > 15 years of experience at a single institution.

**Fig. 1:**
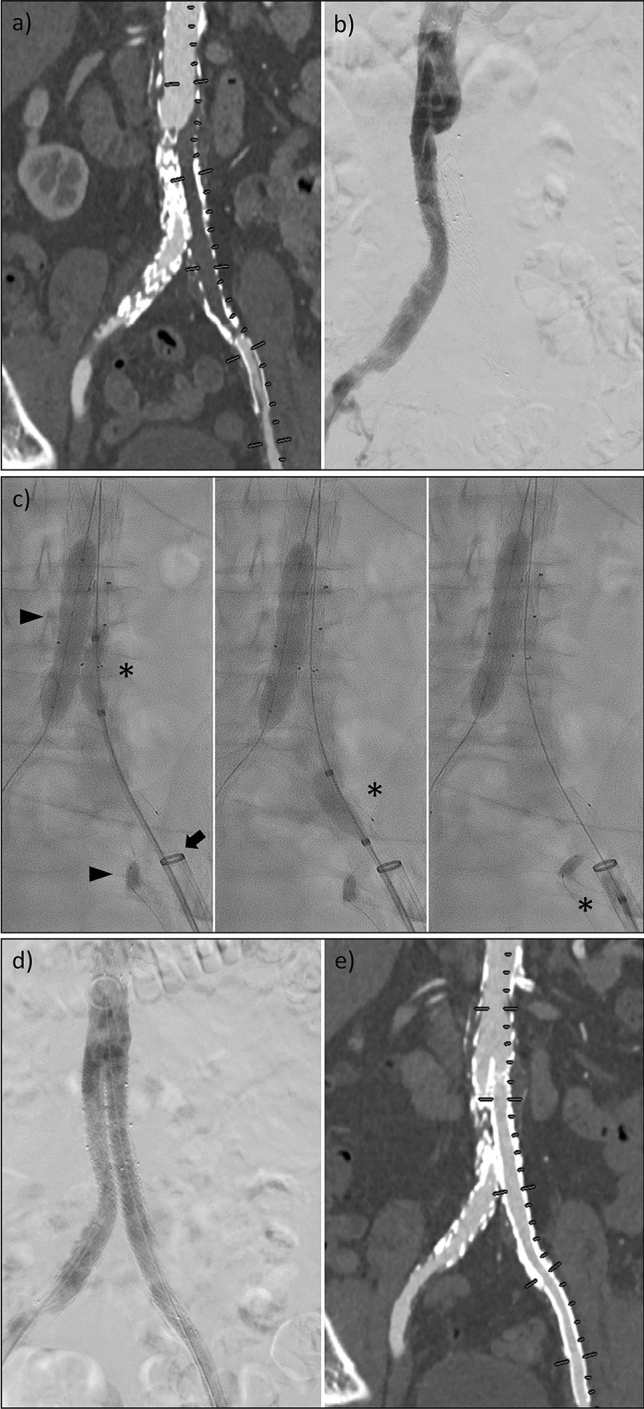
Example of a patient with an occlusion of an iliac limb after EVAR procedure. (**a**) and (**b**) illustrate the initial findings in a CTA maximum intensity projection and digital subtraction angiography, respectively. (**c**) shows the percutaneous thrombectomy. A compliant balloon (*) is continuously pulled into a large-volume sheath placed directly on the occluded prosthesis. For embolic protection, non-compliant balloons were deflated in the adjacent vessels (triangles). (**d**) and (**e**) depict the successful result after the manoeuvre was performed three times and another stent-graft prosthesis was implanted over the distal landing zone of the left iliac artery due to an edge stenosis

**Fig. 2:**
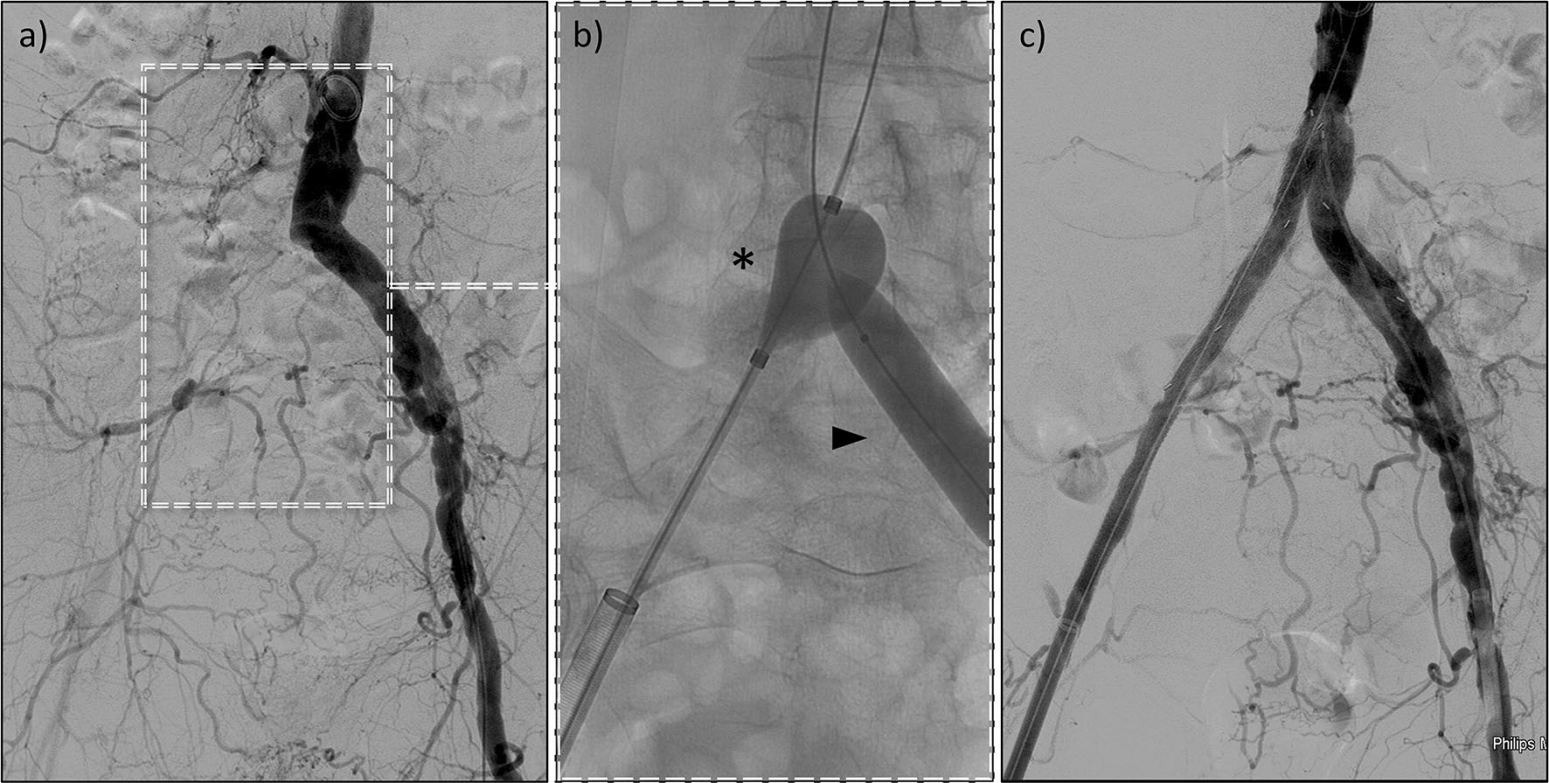
Example of a case with an occlusion of the right iliac artery with extensive atherosclerosis. (**a**) shows the initial findings in subtraction angiography, (**b**) depicts the percutaneous thrombectomy. A compliant balloon (*) was placed proximal to the occlusion. The opposite side was protected from embolism by a non-compliant balloon (triangle). (**c**) illustrates the final result with successful revascularization

The procedure was performed under local anaesthesia. Heparin (5000 International Units) was routinely administered at the start of the intervention. After the successful intraluminal passage of the occluded aortoiliac segment, the puncture site was dilated to 9 French (F), followed by two percutaneous suture-mediated closure systems (Perclose Proglide, Abbott Cardiovascular, Chicago, USA) in the so-called perclose technique. Next, a dedicated large-diameter sheath of 12–24 F (DrySeal, Gore, Flagstaff, USA) was inserted, adapted to the diameter of the access vessel as measured on pre-interventional CT angiography (CTA) images. The sheath size was chosen to occlude the vessel and prevent peripheral embolism. The sheath tip was placed adjacent to the distal end of the occluded vessel segment. The occlusion was then passed with a compliant balloon (46 mm Reliant Stent-graft Balloon, Medtronic, Dublin, Ireland), while a non-compliant balloon (8–12 mm Charger Balloon, Boston Scientific, Malborough, USA) was placed and inflated on the contralateral side for emboli protection (Figs. 1 and 2). The compliant balloon was inflated proximal to the thrombotic segment in the perfused vessel and then gently retracted through the thrombotic occlusion to deliver the thrombus into the sheath. Essentially, the latex-balloon-mediated valve of the dedicated DrySeal sheath must be opened for the declotting retraction manoeuvre, enabling clot removal equivalent to regular FM. The DrySeal sheath is reoccluded immediately after complete retraction of the compliant balloon, as arterial blood flow from the revascularized aortoiliac segment occurs in cases of successful declotting. The procedure was repeated if a thrombus remained in the vessel. If percutaneous thrombectomy alone could not establish reperfusion without residual stenosis < 30%, stent or stent-graft placement was performed. The puncture sides were sealed using the preplaced suture system. After the intervention, the patients were restricted to bed rest until the next day and were monitored for at least 24 h.

### Technical Success and Complications

The criterion for a technically successful intervention was reperfusion of the vessel without relevant residual thrombus or stenosis (< 30%). Primary technical success was assessed directly after percutaneous thrombectomy along with assisted primary technical success if further procedures (e.g., stent placement) were necessary. All patients underwent follow-up CTA or colour-coded duplex sonography within a maximum of three days after the intervention.

The devices used and the mismatch between the large-diameter sheath and the target vessel were recorded. Complications during the intervention, measures taken to treat the complications, and the related success/failure rates were documented. It was determined whether peripheral emboli were significantly dependent on the length of the lesion and the mismatch in the diameter of the inserted sheath and the target vessel. In addition, the length of hospital stay of the patients until discharge from the hospital was recorded.

### Follow Up

After six months, routine follow-up with physical examination was scheduled at an outpatient clinic of the patient's choice. All patients in the stent-graft group underwent follow-up CTA imaging as part of their regular follow-up care. Further diagnostics and imaging in the native-vessel group were performed if the treating physician deemed it necessary. The time intervals and outcomes of these examinations were reported. In addition, all patients were contacted via telephone and enquired regarding any occurrence of symptoms suspicious of re-occlusion or treatment related to re-occlusion since the intervention.

### Statistics

Statistical analyses were performed using the SPSS Statistics software (version 25.0, IBM, USA).

Data were tested for normal distribution using the Shapiro–Wilk test. Parametric data are reported as means and standard deviations, and nonparametric data as medians with 25 and 75% percentiles. Data between groups were tested for significant differences using Student’s t-test for parametric data and Mann–Whitney U tests for nonparametric data. Categorical data were analysed using the *χ*2 test. A Kaplan–Meier curve was computed, and the Mantel Cox test was performed to assess patency rates between patients regarding all follow-up data.

## Results

### Study Population

An overview of the demographics and characteristics of the study population is presented in Table 1.

**Table 1:** Demographic characteristics of the patient collective with pre-existing conditions and the degree of vessel calcifications

	All	Stent-graft	Native vessel	*P*
Number	18	10	8	–
Age [years]	67 ± 9	70 ± 8	63 ± 8	.07
Sex [m/f]	9/8	7/3	3/5	.17
Body mass index [kg/m^2^]	26.5	26.9	26.0	.68
Pre-existing conditions [%]				
Arterial hypertension	94	90	100	.30
Coronary artery disease	39	30	50	.36
Cardiac arrhythmia	22	20	25	.80
Stroke	11	20	0	.18
Peripheral artery disease	56	70	38	.17
Renal insufficiency	50	50	50	.61
Diabetes	17	30	0	.09
Smoking	72	80	63	.41
*Vessel calcification [%]*				
None	0	0	0	.86
Moderate	39	30	50	
Severe	61	70	50	
*Lesion age [%]*				
Acute (< 2 weeks)	22	20	25	.68
Subacute (2–4 weeks)	56	50	63	
Chronic (> 4 weeks)	22	30	13	

Statistical differences are given for the comparison between the two subgroups. A *P* value of < .05 was considered statistically significant. Continuous values are given as mean ± standard deviation

Of the 18 patients identified, ten patients presented with thrombosis related to stent-grafts, and eight with thrombosis in native vessels. A total of *N* = 21 vascular occlusions were treated with percutaneous thrombectomy. All patients underwent CTA of the abdomen and lower limbs to establish a diagnosis before the intervention. There was no significant difference in lesion length or vessel calcification between the groups or in complications.

### Occlusion in Stent-Grafts

A detailed overview of each patient, including the age of the lesion, is presented in Table 2.

**Table 2:** Overview of all patients in the stent-graft group

	Lesion age	Localization			Sheath size [French]	Primary technical success	Secondary technical success	Supplemental procedures
		Aorta	AIC	AIE				
Stent-graft	Subacute	x		x	24		x	Stent-graft
	Acute	x	x	x	24		x	Stent-graft
	Chronic	x	x		20		x	Stent-graft
	Chronic	x	x	x	20		x	Stent-graft
	Chronic	x	x		18	x		Stent-graft
	Subacute		x	x	12		x	Stent-graft
	Subacute	x	x	x	12	x		Stent-graft
	Subacute			x	24	x		Stent-graft
	Acute	x	x	x	20	x		Stent-graft
	Subacute	x	x		12		x	Selfexp. stent

*AIC*   common iliac artery, *AIE*   external iliac artery

Of the ten patients with stent-graft thrombosis, seven (70%) were previously treated with aorto-biiliac stent-grafts for infrarenal aortic aneurysm repair, and three patients (30%) with an iliac stent-graft for common iliac artery aneurysm. Vessel occlusions were localised in the aortoiliac segment in eight patients (80%) and iliac segment in two patients (20%). All lesions were classified as TASC (Inter-Society Consensus for the Management of Peripheral Arterial Disease) type D. The mean occlusion length was 147 ± 4 mm. Two patients with acute onset of symptoms presented with acute limb ischaemia (ALI) category IIa according to the Rutherford classification for ALI. Of the patients with subacute and chronic occlusions, six had severe intermittent claudication (Rutherford category 3 for chronic limb ischaemia [CLI]) and two had rest pain (category 4). The mean ankle-brachial index was 0.61 before treatment.

### Occlusion in Native Vessels

A detailed overview of each patient is presented in Table 3.

**Table 3:** Overview of all patients in the “native vessel” group

	Lesion age	Localization				Sheath size [French]	Primary technical success	Secondary technical success	Supplemental procedures
		Aorta	AIC	AIE	Other				
Native vessel	Subacute		x	x		20		x	Stent-graft
	Subacute	x	x			22		x	EVAR
	Chronic	x				24		x	EVAR
	Subacute	x	x	x		18		x	
	Subacute				x	18	x		
	Acute	x				22	x		
	Subacute		x	x		18		x	Stent-graft
	Acute				x	20	x		

*AIC*   common iliac artery, *AIE*   external iliac artery, *EVAR*  Endovascular aneurysm repair

Among the eight patients with native vessel occlusions, one aortic (13%), two aortoiliac (25%), and five iliac (62%) occlusions were found. All lesions were classified as TASC type D. The average occlusion length was 99  ±  49 mm (*P* = 0.06, compared to the stent-graft group).The two patients with acute onset of symptoms presented with ALI category IIa, according to the Rutherford classification for ALI. Of the patients with subacute and chronic occlusions, four had severe intermittent claudication (Rutherford category 3 for CLI) and two patients had rest pain (category 4). The mean ankle-brachial index before treatment was 0.55 (*P* = 0.76, compared to the stent-graft group).

### Technical Success and Complications

The overall technical success rate was 100%, with primary technical success in 38% (7/18) of the cases (stent-graft group, 40% (4/10); native vessel group, 38% (3/8); *P* = 0.91). In all patients in the stent graft group, stent or stent-graft placement was performed after thrombectomy, while patients in the native vessel group received a stent-graft/EVAR in four cases (50%; 4/8; *P* = 0.01). Reasons for stent (-graft) placement were residual stenosis of > 30% (28%; 5/18), residual thrombus (34%; 6/18), and dissection (17%; 3/18). No amputations or deaths occurred due to this procedure. Further procedural details for each patient are presented in Tables 2 and 3.

Table 4 provides an overview of the complications. The most common complication was peripheral embolism, with 44% (8/18; stent-graft group, 5/10; native vessel group, 3/8). In both groups, embolism was treated successfully via aspiration thrombectomy in the same procedure, although it was supported by local lysis therapy in two cases in the native vessel group (5 mg Actilyse; Boehringer, Ingelheim, Germany). If there was a mismatch of > 1 mm between the diameters of the sheath and target vessel (6/8), peripheral embolism always occurred (*P* < 0.001). There was no significant difference between the lesion length and the occurrence of peripheral embolism (*P* = 0.74).

**Table 4:** Overview of complications of all patients treated with the percutaneous thrombectomy

Procedural complications	All		Stent-graft		Native vessel		*P*
	Number of cases	[%]	Number of cases	[%]	Number of cases	[%]	
Dissection	3	17	2	20	1	13	.68
Peripheral Emboly	8	44	5	50	3	38	.59
Minor (= 1 vessel)	4	22	3	30	1	13	.38
Major (> 1 vessel)	4	22	2	20	2	25	.80
Access side hematoma	5	28	3	30	2	25	.81
Minor (conservative treatment)	5	28	3	30	2	25	.81
Major (surgical treatment)	0	0	0	0	0	0	–
Closure device ass. stenosis	1	5	1	10	0	0	.35

A *P* value of < .05 was considered statistically significant

The median length of hospital stay was 3 [2–6, 9] days, with no significant difference between the two groups (*P* = 0.27).

### Follow Up

Two early re-occlusions occurred in the stent-graft group, which were subsequently treated with bypass surgery.

All patients in the stent-graft group underwent follow-up CTA imaging (219 ± 125 days). In the native vessel group, follow-up CTA was performed in three cases (after 511, 458, and 301 days, *P * = 0.05). No re-occlusions were detected during these follow-up examinations.

**Fig. 3:**
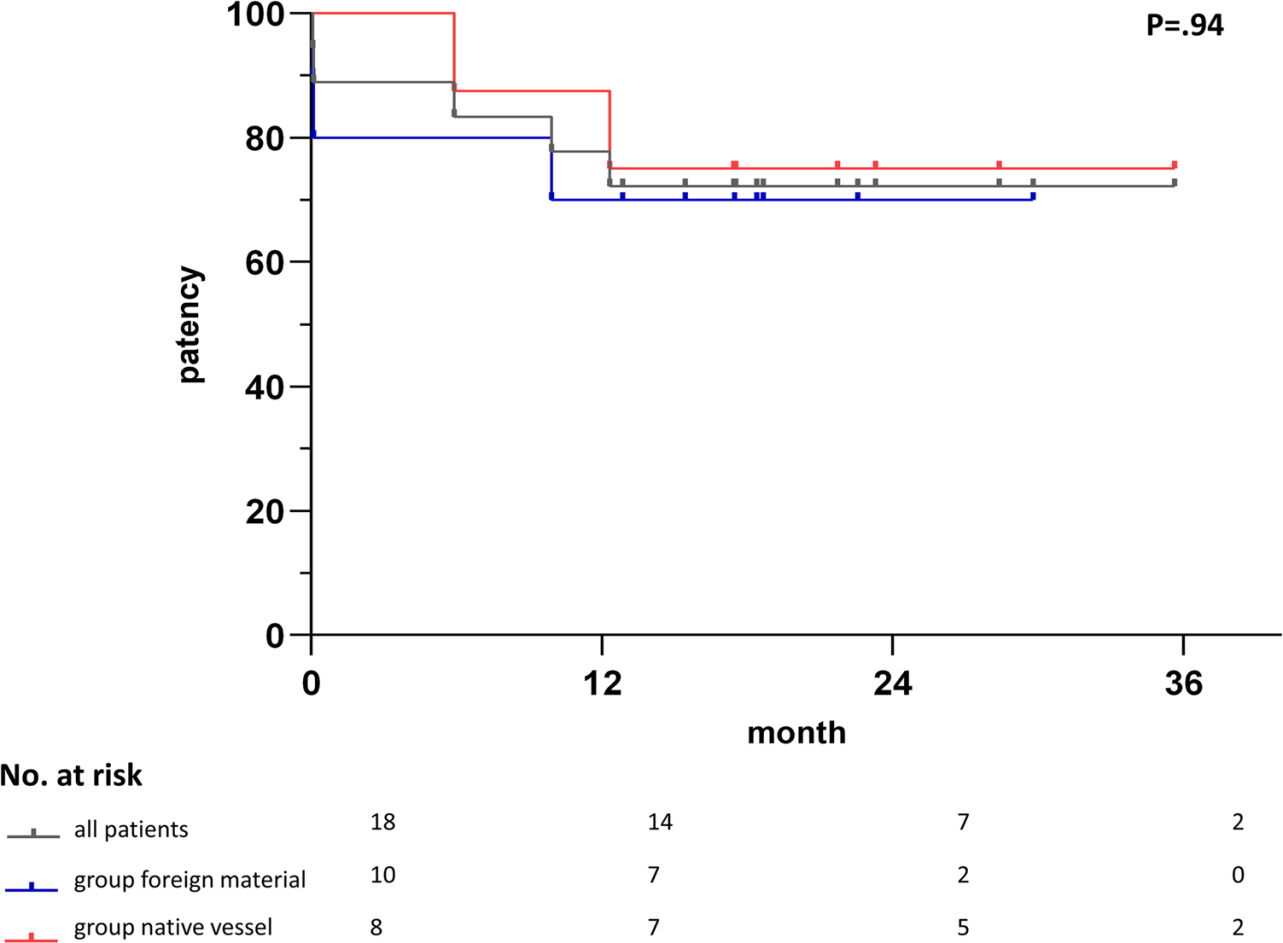
Presentation of the arterial patency following percutaneous thrombectomy of the entire study population (dark grey), the “stent-graft” (red) and the “native vessel” (blue) groups. There was no significant difference between groups. The standard error (Greenwood formula) exceeded 10% for the entire study population (dark grey) after 12 months, for the “stent-graft” (red) group after six months and for the “native vessel” (blue) group after one month

All patients were contacted by phone (follow-up period for all patients, 653 ± 264 days). The follow-up period for patients in the stent-graft group was 538 [443; 589] days; for patients in the native vessel group 796 [598; 960] days (*P* = 0.04). One patient in the stent-graft group reported re-occlusion after 298 days, and two patients in the native vessel group reported re-occlusion after 177 and 372 days. Accordingly, the primary patency after the follow-up period was 72% overall (stent-graft group, 70%; native vessel group, 75%; *P* = 0.94). No deaths or limb loss did occur. Data is illustrated in Fig. 3.

## Discussion

In our case series, percutaneous thrombectomy was successfully performed, the complications that occurred were manageable, and the documented follow-up results were comparable to those of the established techniques.

Empirical data on the endovascular treatment of occlusions in the aortoiliac domain outside of individual case reports are relatively scarce [15]. Cochennec et al. reported a larger cohort of patients with limb graft occlusions, of which only nine (27.2%) were treated using endovascular therapy schemes (three with unspecified thrombectomy + stent [9.1%], six with thrombolysis + stent [18.2%]). Two of these patients experienced graft limb re-occlusion (22.2%) [12]. Ozkan et al. reported five patients with aortoiliac occlusion who were treated with primary aspiration thrombectomy and secondary stenting, achieving an initial success rate and primary patency of 80% after 24 months [13]. Data regarding the outcomes of thrombectomy procedures in peripheral limb ischaemia are more profound. The initial clinical success ranges from 88 to 92.2% for mechanical thrombectomy [4, 16–18] to 83–100% for rheolytic thrombectomy [7, 19]. De Donato et al. reported a primary patency rate of 77.5%/70.4% after 12/24 months for traditional surgical FM, and improved results of 94.0%/87.1% if additional angiography was performed [20]. Considering the challenging vascular territory and high thrombus burden in our case series, the results of the presented percutaneous thrombectomy approach can compete with these established methods with a primary patency rate of 72%.

Several key characteristics, both beneficial and detrimental, of percutaneous thrombectomy in the aortoiliac segment should be addressed. One of the key strengths of this approach is its ability to rapidly remove large volumes of thrombus from the vessel and quickly establish reperfusion which may be beneficial for patient prognosis. Necessary devices such as adequately sized sheaths and balloon catheters are readily available in dedicated departments without the need for additional cost-intensive equipment. Moreover, the diameter of the balloon can be adjusted to the target vessel, whereas the size of the mechanical and rheolytic devices limit their applicability to larger vessels [9]. Furthermore, the percutaneous thrombectomy can be performed regardless of the occlusion acuity.

Compared with traditional FM, this procedure has significant advantages. The benefits of hybrid procedures with parallel angiography have been elaborated in detail in the literature [20, 21]. In addition to peripheral embolism, vascular injuries promote early re-occlusion. The shear force applied by the balloon catheter to the vessel wall correlates with the degree of intimal injury. Thus, the size and compliance of the balloon should be chosen carefully. During the procedure of percutaneous thrombectomy, potential arterial wall injury may not only be detected, but also treated immediately. No open surgical access to the artery is necessary, making wound-healing complications and infections unlikely. Furthermore, general anaesthesia was avoided in all our patients, and the median length of stay until discharge was relatively low at 3 [2–6, 9] days.

Regardless of the advantages outlined above, interventionalists must be aware of vital aspects that may jeopardise clinical success and outcome. The embolism rate for traditional FM has been reported to range from 36 to 86% [20]. In our study, peripheral embolism occurred in 44% of the patients, most likely due to the mismatch of the dedicated large-diameter sheath and the target vessel, resulting in insufficient embolism protection. To prevent embolism, we blocked the branching vessels at risk, such as the contralateral common iliac artery, with balloon occlusion during the declotting manoeuvre, as illustrated in Figs. 1 and 2. Nevertheless, ipsilateral embolism still occurred, and an interventionalist must be capable of treating this complication. In the cases presented here, traditional aspiration thrombectomy, sometimes supplemented with local thrombolysis therapy, was sufficient for this purpose. However, these additional measures must be considered in terms of cost-effectiveness.

Interestingly, the risk of peripheral embolism did not increase with high thrombus burden (*P* = 0.74). However, the main factor in reducing the peripheral embolism rate in our study was optimal sizing of the dedicated large-diameter sheath to the diameter of the target vessel. Specially manufactured devices that can temporarily occlude the vessel, such as sheaths with a compliance balloon at the tip, could solve this problem and improve complication rates. Embolism occurred in two cases, although the sizing of the sheath was adequate in relation to the target vessel. Both the patients presented with a high degree of atheromatous atherosclerosis, and therefore, embolism may have already occurred during sheath placement before the thrombectomy manoeuvre. Nevertheless, the origin of embolism in these cases remains speculative.

## Limitations

There are several limitations of the study that should be addressed:

It was a retrospective analysis of a relatively small patient population, thereby inheriting the characteristic limitations of a retrospective study. Therefore, the presented results may be viewed as a pilot study demonstrating the general feasibility and the first evidence of suitable indications for percutaneous thrombectomy. Patient follow-up was not standardised, and the time intervals varied accordingly. Thus, long-term success rates, both technical and clinical, and data regarding secondary patency could not be obtained. We did not directly compare patients treated with percutaneous thrombectomy, traditional FM, or other endovascular thrombectomy procedures for the aortoiliac segment. The decision to perform percutaneous thrombectomy was made on a case-to-case basis.

## Conclusion

Percutaneous thrombectomy is a promising approach to declotting the aortoiliac segment. It offers various advantages, such as performance under local anaesthesia and early discharge, and can be successfully applied to the pathological spectrum presented here. However, the interventionalist should be capable of treating typical complications such as peripheral embolism. Finally, this encouraging early report emphasises the need for further exploration of this promising technique for treating challenging clinical dilemmas.

## References

[1] Fogarty TJ, Cranley JJ, Krause RJ (1963). A method for extraction of arterial emboli and thrombi. Surg Gynecol Obstet.

[2] Plecha FR, Pories WJ (1972). Intraoperative angiography in the immediate assessment of arterial reconstruction. Arch Surg.

[3] White GH, White RA, Kopchok GE (1988). Angioscopic thromboembolectomy: preliminary observations with a recent technique. J Vasc Surg.

[4] Heller S, Lubanda JC, Varejka P (2017). Percutaneous mechanical thrombectomy using Rotarex(R) S device in acute limb ischemia in infrainguinal occlusions. Biomed Res Int.

[5] Zeller T, Frank U, Burgelin K (2003). Early experience with a rotational thrombectomy device for treatment of acute and subacute infra-aortic arterial occlusions. J Endovasc Ther.

[6] Alexopoulos D, Davlouros PA (2012). Thrombus extraction catheters vs. angiojet rheolytic thrombectomy in thrombotic lesions/SV grafts. Curr Cardiol Rev.

[7] Ierardi AM, Xhepa G, Piffaretti G (2015). Clinical experience with Angiojet: a comprehensive review. Int Angiol.

[8] Carrera LA, Reddy R, Pamoukian VN (2013). Massive intravascular hemolysis with mechanical rheolytic thrombectomy of a hemodialysis arteriovenous fistula. Semin Dial.

[9] de Donato G, Pasqui E, Setacci F (2018). Acute on chronic limb ischemia: from surgical embolectomy and thrombolysis to endovascular options. Semin Vasc Surg.

[10] Izaki K, Yamaguchi M, Matsumoto I (2011). Percutaneous selective embolectomy using a Fogarty Thru-Lumen Catheter for pancreas graft thrombosis: a case report. Cardiovasc Intervent Radiol.

[11] Nishimoto Y, Toma M, Miyamoto R (2021). Successful percutaneous fogarty arterial thrombectomy for acute lower limb ischemia. JACC Cardiovasc Interv.

[12] Cochennec F, Becquemin JP, Desgranges P (2007). Limb graft occlusion following EVAR: clinical pattern, outcomes and predictive factors of occurrence. Eur J Vasc Endovasc Surg.

[13] Ozkan U, Oguzkurt L, Tercan F (2009). Endovascular treatment strategies in aortoiliac occlusion. Cardiovasc Intervent Radiol.

[14] Sieren MM, Widmann C, Weiss N (2021). Automated segmentation and quantification of the healthy and diseased aorta in CT angiographies using a dedicated deep learning approach. Eur Radiol.

[15] Uotani K, Hamanaka A, Matsushiro K (2018). Endovascular aneurysm repair with balloon thrombectomy for acute thrombosis of abdominal aortic aneurysm. Cardiovasc Intervent Radiol.

[16] Lagana D, Carrafiello G, Lumia D (2011). Recanalisation of thrombotic arterial occlusions with rotational thrombectomy. Radiol Med.

[17] Stahlberg E, Anton S, Sieren M (2021). Mechanical rotational thrombectomy in long femoropopliteal artery and bypass occlusions: risk factors for periprocedural peripheral embolization. Diagn Interv Radiol.

[18] Fluck F, Stephan M, Augustin A (2021). Percutaneous mechanical thrombectomy in acute and subacute lower-extremity ischemia: impact of adjunctive, solely nonthrombolytic endovascular procedures. Diagn Interv Radiol.

[19] Leung DA, Blitz LR, Nelson T (2015). Rheolytic pharmacomechanical thrombectomy for the management of acute limb ischemia: results from the PEARL registry. J Endovasc Ther.

[20] de Donato G, Setacci F, Sirignano P (2014). The combination of surgical embolectomy and endovascular techniques may improve outcomes of patients with acute lower limb ischemia. J Vasc Surg.

[21] Setacci C, De Donato G, Setacci F (2012). Hybrid procedures for acute limb ischemia. J Cardiovasc Surg (Torino).

